# Advance Health Care Directives and “Public Guardian”: The Italian Supreme Court Requests the Status of Current and Not Future Inability

**DOI:** 10.1155/2014/576391

**Published:** 2014-03-05

**Authors:** Francesco Paolo Busardò, Stefania Bello, Matteo Gulino, Simona Zaami, Paola Frati

**Affiliations:** ^1^Department of Anatomical, Histological, Forensic and Orthopaedic Sciences, University of Rome La Sapienza, Viale Regina Elena 336, 00161 Rome, Italy; ^2^Department of Legal Medicine, University of Foggia, Via degli Aviatori, 71121 Foggia, Italy; ^3^Neuromed, Istituto Mediterraneo Neurologico (IRCCS), Via Atinense 18, Pozzilli, 86077 Isernia, Italy

## Abstract

Advance health care decisions animate an intense debate in several European countries, which started more than 20 years ago in the USA and led to the adoption of different rules, based on the diverse legal, sociocultural and philosophical traditions of each society. In Italy, the controversial issue of advance directives and end of life's rights, in the absence of a clear and comprehensive legislation, has been over time a subject of interest of the Supreme Court. Since 2004 a law introduced the “Public Guardian,” aiming to provide an instrument of assistance to the person lacking in autonomy because of an illness or incapacity. Recently, this critical issue has once again been brought to the interest of the Supreme Court, which passed a judgment trying to clarify the legislative application of the appointment of the Guardian in the field of advance directives.

## 1. Introduction

Advance health care decisions animate an intense debate in several European countries, which started more than 20 years ago in the USA and led to the adoption of different rules, based on the diverse legal, sociocultural, religious, and philosophical traditions of each society [1].

In Italy, the controversial issue of end of life rights, in the absence of a clear and comprehensive legislation, has been once again brought to the attention of the Supreme Court which passed the judgment no. 23707 of December 20, 2012 [2], trying to clarify the legislative application of the appointment of the Guardian in the field of advance directives. The Supreme Court has established that only if the subject is in a state of full capacity can he proceed to the designation of a Guardian, but its appointment by the Court and the occurrence of the effects should be postponed until the onset of a condition of incapacity or infirmity occurs. According to the Judges, the appointment of a Guardian by a public or private deed remains confined within the framework of a private initiative, the effects of which have exclusively a private value since it does not postulate the intervention of the Court [2]. The Supreme Court has therefore clearly pronounced on the necessity of the requirement of a conjunction between the process of appointing the Guardian and the onset of the pathological condition. The Rule on the appointment of the Guardian introduced by Law no. 6 of 2004 [3], as stated by this Court with judgment no. 13584/2006, aims to provide an instrument of assistance to the person lacking in autonomy because of an illness or incapacity, and therefore the judge with respect to the degree of intensity of this situation excludes the more invasive procedures of traditional institutions aimed at protecting incompetents, such as interdiction and incapacitation. Consequently the judicial intervention, consistent with this purpose, can only be contextual to the manifestation of the need to protect the subject, which is the assumption of the same institute and not only of its effects. According to section 407 of Civil Code and 720 *bis* of Code of Criminal Procedure, the judicial intervention is placed “rebus sic stantibus” (Latin for “things thus standing”), and for this reason the adoption of the Guardian “now for later” is not allowed, in view of a future condition [3].

The first aim of this paper is to review the Italian legislative and jurisprudential evolution in the field of advance health care directives, taking into account the recent judgment of the Supreme Court (no. 23707 of 20 December 2012), which has had an impact on the controversial issue of end of life rights and the Guardian's appointment. Secondly, the Authors compared the Italian legislation in this field with other European countries.

## 2. The Guardian, Medical Treatments, and Advance Health Care Directives in Italy

The legislator with the law 6/2004 [3] has introduced the legal status of the Guardian, denying the rigid and schematic logic of the old forms of protection of the incapacitated person, where very little space was left to the residual freedom of the individual, mortifying his dignity. The aim of the new institution is a flexible and adaptable modulation to the needs of protection to safeguard the whole panorama of human frailty, “an individual and modular protection, as a result of a conception of the rights of vulnerable subjects, which complies with the provisions of the Constitution that aims to promote the full development of the human person.”

The legislative process in the field of end of life decisions saw already in 1984 a draft law (by Loris Fortuna) to regulate the interruption of treatment for the terminally ill. This draft law was followed by others: on February 10, 1999, a legislative proposal (no. 5673) was presented by 16 members of the Italian parliament: “provisions on informed consent and declarations on advance health care directives”; on June 29, 2000, some Senators of the Green party presented another draft law (no. 4694) on the same matter and finally, on January 9, 2004, the present law (6/2004) was enacted [3]. The adaptability of the contents of the Guardian figure introduced by Law 6/2004 and the speed of the procedure of its appointment made it as a useful tool used for any type of disability, in respect of the person. This approach has meant that in a short time it spread also among medical treatments. It is supported by article 404 of Civil Code and article 1 of the law 6/2004 whose purpose is to protect with the least possible limitation those subjects without a total or partial autonomy. This process has contributed to the rapid spread of the Guardian figure in healthcare. Initially, frail patients have activated the procedure of Guardian appointment to find comfort in someone of trust, in case it would have been necessary to choose the most appropriate medical treatment. Soon after, the impossibility of subjects to project their will in future perspective due to the lack of a specific law on advance health care directives brought to rapidly spread the practise of resorting the Guardian to ensure the respect of their own will once they become incapacitated. In this perspective the Italian judiciary began to accept the requests of advance health care directives (Court of Cosenza 24/10/04, Court of Turin 26/2/07, Court of Vibo Valentia 30/11/05, Court of Modena 15/9/04, Court of Rome 28/1/05, and Court of Trieste 20/11/08) taking into account the right of self-determination of the patient, stating that it is useful to use it in cases of partial or complete cognitive impairment. With a reference to Oviedo Convention of April 4, 1997, [4] the judgments above identified “the Guardianship as an expression of the right of therapeutic self-determination of the patient, which has to be exercised within a framework of therapeutic alliance with the doctor.”

In case of refusal of care and advance directives, the judiciary has proved to be less concordant; among reasons that have led the judiciary to recognize the value of advance directives carried out by Guardian's appointment, the following aspects can be highlighted: the duty to respect the will of the applicant that is based on the guarantee of the fundamental rights of freedom and dignity of the person protected by the Italian Constitution (Articles 2, 3, and 13) and the possibility of nonactuality of the manifestation of the will compared to the onset of the disease. From the other side, the most common reason for which people have required the Guardian's appointments can be resumed as follows:

- to enforce the decision of the patient in the event of loss of consciousness (typical is the case of the refusal of blood transfusion by Jehovah's Witnesses: Court of ViboValentia 30/11/2005, Court of Rome 20/12/2005, Court of Treviso 9/2/2006, Court of Modena 16/9/2008, Court of Bari 18/8/2011, and Supreme Court no. 23707 of December 20, 2012);
- when a subject is undergoing a surgery and through the Guardian's appointment wishes to express the desire not to be subjected to medical treatment intended to prolong an irreversible state (Court of Reggio Emilia 04.11.05, Court of Modena 13/5/2008, Court of Modena 23/12/2008, and Court of Modena 27/7/2012);
- for subjects without existing health problems but intending to enforce their will in the future. For the latter occurrence there were favourable judgements (Court of Modena 5/11/08, Court of Parma 2.4.04, Court of Rome 19/3/04, etc.) and negative judgements (Court of Rome 1/4/09, Court of Pistoia 1/4/09, Court of Genoa 6/3/09, Court of Cagliari 14/12/09, etc.).

The recent Judgment no. 23707/12 of the Supreme Court [2] although from one side has clarified a conflict of the Italian jurisprudence, which led to decide the same situations in different ways, from the other one it stated the requirement of conjunction between the process of Guardian's appointment and the onset of a pathological condition. The Supreme Court in the Judgment above mentioned rejected the appeal of a woman, who requested the appointment of a Guardian for a future and merely possible loss of capacity. The woman, in her full physical and mental capabilities, had notarized a private agreement by which she nominated a Guardian, stating her willingness about medical treatments which may or may not be subjected in the future and she gave the Guardian full decision-making in this regard. The tutelary judge of Trento had declared the inadmissibility of the request of Guardian's appointment and the Court of Appeal had rejected the claim based on the assumption that such a request could not come from the person in full possession of his/her mental and physical capabilities. The Supreme Court agreed with the lower Courts that the activation of the procedure for the Guardian's appointment should be conditional upon the occurrence of the condition and the onset of illness or incapacity “which was inspired by the need to protect the ratio of the institution under discussion.” The right attributed by art. 408 of Civil Code to designate the Guardian for the future would produce its effects only on a private level, not postulating the intervention of the Court, consequently the preventive designation aims to enhance the relationship of trust between the appointer and the person chosen. The Supreme Court states that the act of designation will bind the Guardian's appointment, although its powers are not fixed but set by the tutelary judge in exercising its decision-making power for pursuing the objectives of “care” necessary to ensure the protection of the beneficiary. The effects of designation, however, require the appointment by the tutelary judge of the designated person, which is possible only when the condition of incapacity or infirmity has become current.

## 3. Advance Heath Care Directives in Europe: An Overview

The possibility to express, in a state of well-being and capacity, the will about any medical treatment for a long time has animated the political and scientific debate with important consequences in terms of legislation and jurisprudence. In Italy, differently from other European countries, the only possibility of advance directive is the one made by the organ donation. This divergence raises many doubts, especially when compared with the international trend and with the legislation of other European countries, and aims to recognize the validity of these documents. In France, law 2005-370 of 22 April 2005 [5], established that every adult can formulate advance directives (directives anticipées), which are valid for three years and that can always be revoked, and they can be used in the event of a state of incapacity, containing information about the type of treatment as to interrupt. The later Decree no. 2006-119 of 6 February 2006 [6] amended the Code de la Sanite publique and ruled that the directives need to be written, dated, and signed by the author with an explanation of his personal details. In Germany, the law on advance directives from 1 September 2009 [7] stated that they must be respected in any decision concerning medical treatment, regardless of the stage of the illness. It can be informally revoked at any time, even with limited decision-making capacity. Nobody may be obliged to issue a directive in any way. In addition, it is expected that in the absence of a written declaration, evidential value to the reconstruction of the ethical and religious convictions of the interested party should be given. In Spain, the law no. 41 of 14 November 2002 [8] requires that advance health care directives have to be written, prohibiting their applicability if in direct conflict with the general principles of law and good medical practice.

In UK, the Mental Capacity Act came into force on October 1, 2007 [9]. The Act makes provision for people to plan ahead for a time when they can need help. This introduces advanced decisions to refuse treatment. The Act upholds the principle of Best Interest for the individual concerned.

A Court of Protection is able to give support for difficult decisions. The Office of the Public Guardian (formerly Public Guardianship Office), the administrative arm of the Court of Protection, will help the Act work. The Netherlands Law, adopted in 2001 and in force since 1 April 2002 [10], ruled that patients and prospective patients may specify the circumstances under which they would like to undergo euthanasia. They make this provision by a written euthanasia directive. This helps establish the previously expressed wish of the patient even if he/she is no longer able to communicate. Generally, in Europe there is certain openness in recognizing the advance health care directives, also supported by the statements of international organizations. The Supreme Court in the judgment under discussion tends to emphasize the fact that places the individual as the author of his/her own choices regarding health, enhancing the right of self-determination in both its positive and negative meaning, and the capability to dispose of him/herself preventively and not contextually to the onset of the disease. The judgment also clearly cites both the Charter of Fundamental Rights of the European Union entered into force with the Treaty of Lisbon of 2009 and the Oviedo Convention and the case of Pretty v. United Kingdom (2346/02) decided by European Court of Human Rights in 2002 [11].

## 4. Discussion and Conclusions

The recent Judgment of the Italian Supreme Court represents from one side a useful tool to clarify a dangerous conflict of jurisprudence, which led to decide the same situations in different ways; at the same time it represents a significant setback on the only possibility of operating advance directives in our legal system. *De facto*, presently, the only Italian orientation is once again the Code of medical ethics, which in accordance with the international context previously mentioned, tries to give some dignity to what at European and extra European level has long been shared and accepted. The code, despite not having the force of law, recognizes by art. 38 the fundamental role, although nonbinding, of advance directives “the doctor, if the patient is not able to express his will, must take into account in his choices the willingness expressed by the patient in a previously documented and reliable way” [12]. Another reference to the code of medical ethics in the field of advance heath care directives is provided by the Italian National Federation of the Associations of Physicians and Dentists (FNOMCeO) in a document of 2009 [13]. According to the Code, the principle of guarantee is violated when the doctor, intentionally and with appropriate means, operates to the end of life even if it is requested by the patient (euthanasia) or insists on unnecessary treatments from which an improvement of the disease or the quality of life cannot be expected (therapeutic obstinacy) [13]. The autonomy and responsibility of the physician is to ensure that requests for care of the patients are received in the ongoing effort to help those who suffer and have the right to be accompanied with responsibility and solidarity [14]. Taking into account the provisions of the Italian Supreme Court in the well-known case of “Eluana Englaro”, which allowed in November 2008 the woman's father to suspend the nutrition and hydration given to his daughter Eluana, it is necessary to refer to the principle that the Guardian may ask permission from the judge, once the irreversible nature of the state of unconsciousness and the reconstruction of the will of the person about his beliefs, lifestyle, and overall personality is proven, regarding the interruption of treatments and therapies of life support. Between the lines of the judgment emerges the possibility for the guardian to consent or dissent to medical treatment in accordance with the will of the person.

The multiple measures before the judgment under review approved the appointment of a Guardian in the period before the onset of the condition of incapacity and they were in agreement with the arguments put forward by Judges in the case “Englaro” [15], stressing that the loss of consciousness cannot also result in the loss of fundamental rights and freedoms such as self-determination in personal choices. Only the appointment of the Guardian could rekindle the dialogue between doctor and patient, ensuring respect for the rights of a personal nature. Probably we may have missed an important opportunity to protect the dignity and freedom of the individual [16], placing Italy in countertrend with other European Countries, which attribute a significant value to patient autonomy and to the possibility of making advance health care decisions [17].

The central role of Italian High Court in the open debate of advance health care directives if from one side has the aim to encourage a particular course of action; however judicial pronouncements may not be always the best means to achieve this aim; therefore a thought that is reflected in the physician's daily work is: can doctors learn from judges' decisions [18]?
